# Tirzepatide and Cardiometabolic Effects in Obese Non-diabetic Adults: A Systematic Review, Meta-Analysis, and Narrative Synthesis of Cardiovascular Outcomes

**DOI:** 10.7759/cureus.109350

**Published:** 2026-05-21

**Authors:** Pravalya Chaparala, Dinesh Aravind Rongali, Maha Khot, Brahmaiahchari Rangachari, Kashif Uddin Ahmed, Sparsh Suri, Mothe Greeshma, Muhammad Muddassar Shafiq

**Affiliations:** 1 Medicine, Rush University Medical Center, Chicago, USA; 2 Endocrinology, Dr. D.Y. Patil University School of Medicine, Navi Mumbai, IND; 3 Biomedical Sciences, Kentucky College of Osteopathic Medicine, University of Pikeville, Pikeville, USA; 4 Medicine, Osmania Medical College, Hyderabad, IND; 5 Medicine, Kamineni Institute of Medical Sciences, Narketpally, IND; 6 Medicine, Kakatiya Medical College, Telangana, IND; 7 Internal Medicine, Punjab Rangers Teaching Hospital (PRTH), Lahore, PAK

**Keywords:** cardiometabolic, cardiovascular outcomes, non-diabetic adults, obesity and diabetes, tirzepatide

## Abstract

Obesity is a recurrent disease condition that predisposes individuals to an increased likelihood of developing hypertension, dyslipidemia, insulin resistance, systemic inflammation, heart failure, and atherosclerotic cardiovascular disease (ASCVD). Tirzepatide, a dual agonist of the glucose-dependent insulinotropic polypeptide and glucagon-like peptide-1 receptors, has been shown to produce clinically significant weight loss and metabolic improvements. However, the combined cardiometabolic effects of tirzepatide in adults with obesity or who are overweight and have no diabetes are not fully understood. In this systematic review and meta-analysis, the effects of tirzepatide were systematically evaluated using data from randomized controlled trials in adults with obesity or overweight without diabetes. The meta-analyzed body weight effects of tirzepatide included data from four clinical trials. Weight-loss measures assessed include percentage weight change, percentage change in waist circumference, percentage change in systolic and diastolic blood pressure, and reductions in triglycerides, LDL cholesterol, and HDL cholesterol. Cardiometabolic effects were analyzed using a random-effects generic inverse-variance model. Cardiovascular outcomes based on heart failure with preserved ejection fraction and ASCVD populations were narratively assessed. According to the results of this analysis, the overall estimate for the tirzepatide effect size compared to placebo shows significant weight loss (MD = -18.42%; 95% CI, -22.05 to -14.79; I² = 70.9%). When omitting the randomized-withdrawal study, there was still a substantial effect (MD = -18.01%, 95% CI, -24.86 to -11.16; I² = 77.6%). A highly statistically significant reduction in waist circumference was also observed (MD = -15.20 cm; 95% CI, -17.69 to -12.70; I² = 23.5%). Additionally, significant improvements in blood pressure and lipid measures were shown by tirzepatide. It is worth mentioning that the effect sizes for blood pressure and lipids are less precise due to a smaller sample size. Overall, tirzepatide is effective in producing considerable improvements in body weight and waist circumference in adults with obesity or overweight without diabetes. However, evidence regarding hard cardiovascular outcomes in this population remains insufficient.

## Introduction and background

Obesity is commonly known to be a chronic and recurrent disease that contributes substantially to global morbidity and mortality [1-3]. Its epidemiologic burden has increased markedly over recent decades: the World Health Organization reported that more than 1 billion people were living with obesity in 2022, while adult obesity prevalence more than doubled between 1990 and 2022, and childhood/adolescent obesity quadrupled over the same period. In 2022, approximately 16% of adults worldwide were living with obesity, and 43% of adults were overweight, highlighting the broad population-level scale of excess adiposity. This rising prevalence has major health-system and economic implications because obesity increases the risk of cardiometabolic complications, including type 2 diabetes mellitus (T2DM), hypertension, dyslipidemia, heart failure, and atherosclerotic cardiovascular disease (ASCVD) [3-6]. The financial burden is also substantial; global estimates suggest that the economic impact of overweight and obesity could exceed US$4 trillion annually by 2035 if current trends continue. Therefore, obesity should be viewed not only as an individual clinical condition but also as a major global public health challenge requiring effective long-term prevention and treatment strategies.

It should be noted that obesity can be prevented and managed through lifestyle modification, including dietary and physical activity changes. However, sustaining weight loss is challenging and often requires more than simple diet and exercise [7-9]. The biological, behavioral, environmental, genetic, and social factors underlying obesity make it difficult for many patients to sustain a healthy body weight after initial weight loss. In addition to individual-level factors such as appetite regulation, metabolic adaptation, physical activity, and genetic susceptibility, social determinants of health, including socioeconomic status, education, neighborhood environment, access to healthy foods, healthcare access, chronic stress, and structural inequities, can strongly influence obesity risk and cardiometabolic outcomes. These factors are increasingly recognized in cardiology because they contribute to unequal exposure to cardiovascular risk factors such as obesity, hypertension, diabetes, dyslipidemia, and smoking. Borkowski et al. specifically highlight racial and socioeconomic determinants as important contributors to cardiovascular disease risk and disparities, supporting the need to consider obesity within a broader social and cardiometabolic framework. Therefore, long-term obesity management often requires a comprehensive approach that combines lifestyle modification with pharmacological treatment when clinically appropriate, particularly for patients who have difficulty maintaining weight loss through behavioral intervention alone [10-12].

Tirzepatide is a drug that acts via two incretin targets, glucose-dependent insulinotropic polypeptide and glucagon-like peptide-1 [13-15]. The drug's action results in decreased appetite, reduced energy intake, improved regulation of glucose metabolism, and clinical weight loss. Evidence regarding the effects of tirzepatide in adults with obesity or overweight without diabetes is available based on randomized trials. Treatment with tirzepatide at doses of 5 mg, 10 mg, and 15 mg resulted in significant weight reduction in adults without diabetes (N = 2539) [16]. Another study by Wadden et al. [17] showed that tirzepatide led to additional weight loss in patients after lifestyle modifications. Aronne et al. [18] found that continuous administration of the drug resulted in weight loss maintenance and progression, while cessation of tirzepatide treatment led to weight gain. More recently, Zhao et al. [19] reported findings on tirzepatide-induced weight loss in a Chinese population with obesity or overweight and weight-related comorbidities; both the 10 mg and 15 mg doses resulted in clinically meaningful weight loss.

Although the weight loss effects of tirzepatide have been sufficiently documented, its influence on cardiometabolic and cardiovascular risk markers among patients with obesity but no diabetes should be carefully assessed. Evidence from incretin-based therapy in heart failure with preserved ejection fraction (HFpEF) with obesity and from the SUMMIT tirzepatide program is clinically relevant. Still, these patient populations cannot be applied generally to all patients with obesity [20-22]. Likewise, observational studies among those with established ASCVD without diabetes provide valuable information that cannot be used to draw the same conclusions as randomized placebo-controlled obesity studies [23].

Therefore, a combination of cardiovascular and obesity trials with different designs should be avoided in a meta-analysis. This systematic review and meta-analysis was designed to investigate the effects of tirzepatide on body weight and cardiometabolic risk factors in adults with obesity or overweight without diabetes.

## Review

Methods

Study Design and Reporting Framework

**Figure 1:**
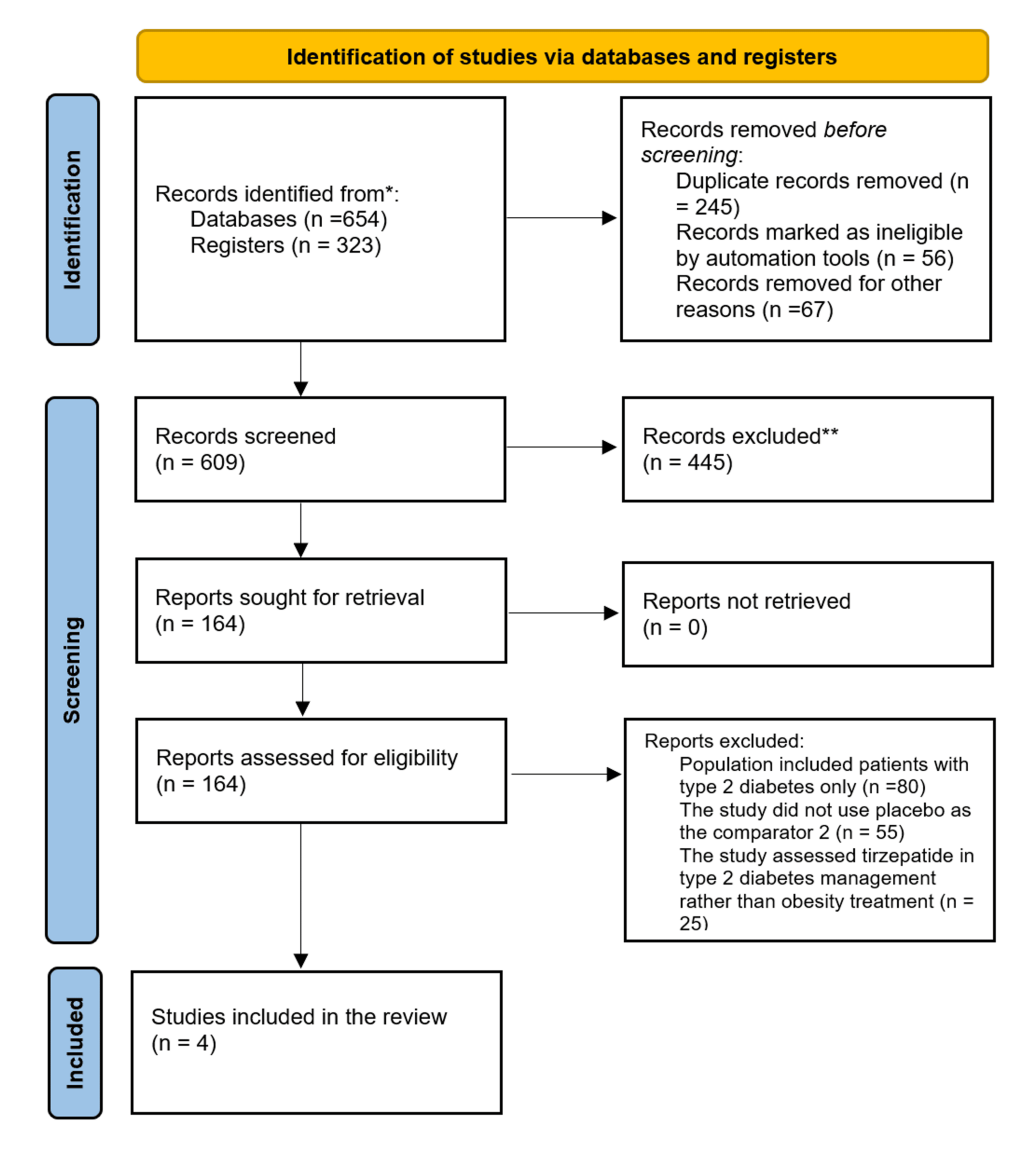
PRISMA flowchart PRISMA: Preferred Reporting Items for Systematic Reviews and Meta-Analyses

The systematic review and meta-analysis were performed following Preferred Reporting Items for Systematic Reviews and Meta-Analyses (PRISMA) guidelines (Figure 1) [24]. The quantitative analysis included randomized controlled trials (RCTs) of tirzepatide in obese or overweight subjects without diabetes mellitus. Cardiovascular endpoints from trials that significantly varied in subject population, study design, or comparator drugs were included in the qualitative analysis only.

PICO Framework

To conduct the primary quantitative meta-analysis, the PICO (population, intervention, comparator, and outcome) criteria, as shown in Table 1, were strictly limited to patients with obesity/overweight without diabetes. Clinical trials involving patients with T2DM were included in the pooled cardiometabolic meta-analysis only if they reported data separately for those without diabetes. Cardiovascular clinical trials involving other patient groups (e.g., HFpEF and/or ASCVD patients) were not pooled with the primary RCTs but were reported narratively.

**Table 1:** PICO framework PICO: population, intervention, comparator, outcome, BMI: body mass index, mg: milligram, LDL: low-density lipoprotein, HDL: high-density lipoprotein, HFpEF: heart failure with preserved ejection fraction, RCTs: randomized controlled trials

PICO element	Definition used in this review
Population	Patients aged ≥18 years who are obese or overweight, either with or without any associated weight-related comorbidity, but not diabetic at baseline. The definition of obesity/overweight varied by study, typically BMI ≥30 kg/m2 or BMI ≥27 kg/m2 with weight-related comorbidity.
Intervention	Once-weekly subcutaneous tirzepatide, including fixed doses of 5 mg, 10 mg, or 15 mg, as well as maximum tolerated dose regimens of 10 mg or 15 mg.
Comparator	Placebo with lifestyle intervention, or placebo following tirzepatide discontinuation, according to the specific trial design.
Primary outcome	Percentage change in body weight from baseline or randomization through the final follow-up assessment.
Secondary outcomes	Waist circumference, systolic blood pressure, diastolic blood pressure, triglyceride levels, LDL cholesterol, HDL cholesterol, and adverse events were available for extraction.
Exploratory cardiovascular outcomes	Cardiovascular mortality, worsening events related to heart failure, heart failure hospitalizations, major adverse cardiovascular events, myocardial infarction, stroke, and all-cause mortality. All of these outcomes were reviewed narratively because the cardiovascular trials had different populations and comparisons.
Study design	RCTs for the primary quantitative meta-analysis. The observational study and the HFpEF-related studies were considered for the narrative cardiovascular analysis alone.

Eligibility Criteria

Studies included in the quantitative meta-analysis needed to fulfill the following inclusion criteria: adult participants, 18 years of age or older; obesity/overweight, with or without obesity-associated comorbidities; diabetes-free subjects at baseline; randomized clinical trials; fixed doses or maximum tolerated dose of tirzepatide; presence of a placebo control; and presence of at least one cardiometabolic outcome that could be extracted from the study.

In addition to these inclusion criteria, the exclusion criteria for the primary meta-analysis would include studies of patients with exclusively T2DM, studies without a placebo, non-RCT studies, review articles, previous meta-analyses, incomplete data from conference abstracts, and studies lacking effect sizes. As for cardiovascular studies on the effects of tirzepatide treatment in obese/overweight patients, only studies with extractable data from 2015 to 2025 were included in the narrative synthesis.

Information Sources and Search Strategy

A search strategy was developed to retrieve RCTs examining the effects of tirzepatide treatment on outcomes among patients with obesity or overweight. The databases used for this search included PubMed/MEDLINE, Google Scholar, Cochrane Library, ClinicalTrials.gov, and references from reviews.

The following keywords were used: "Tirzepatide" OR "LY3298176" OR "Mounjaro" OR "Zepbound" AND "obesity" OR "overweight" OR "weight reduction" OR "weight loss" AND "without diabetes" OR "non-diabetic" OR "excluding diabetes" AND "randomized" OR "placebo" OR "trial" OR "cardiovascular outcomes." For cardiovascular analyses, keywords would be "heart failure" OR "HFpEF" OR "MACE" OR "ASCVD".

Outcomes

The primary outcome measure was the percentage change in body weight. Secondary outcomes included changes in waist circumference, systolic blood pressure, diastolic blood pressure, triglycerides, LDL cholesterol, and HDL cholesterol. Cardiometabolic exploratory outcomes would be cardiovascular death, worsening heart failure, heart failure hospitalizations, major adverse cardiovascular events, myocardial infarction, stroke, and all-cause mortality.

Data Extraction

Data were extracted using author-year labels. Trial names were retained only in the study characteristics table to provide context. For trials with multiple tirzepatide arms, the 15 mg arm was used for the primary analysis to avoid double-counting the placebo group and to represent the highest obesity-relevant dose. For trials using a maximum tolerated dose design, the reported maximum tolerated dose estimate was used.

Extracted variables included author, year, design, population, sample size, intervention dose, comparator, follow-up duration, outcome type, effect estimate, confidence interval, and unit of measurement.

Continuous outcomes reported as treatment differences with 95% confidence intervals were pooled using a generic inverse-variance meta-analysis, following Cochrane guidance and standard meta-analytic implementation in R (R Foundation for Statistical Computing, Vienna, Austria, https://www.R-project.org/) [25,26]. Standard errors were calculated from confidence intervals using:

$SE = \frac{\text{Upper CI} - \text{Lower CI}}{3.92}$

Random-effects models were used because of clinical and methodological heterogeneity across trials, including differences in trial design, follow-up duration, geographical population, dose strategy, and lead-in intervention [25-27]. The Hartung-Knapp adjustment was applied to the random-effects confidence intervals [27]. Heterogeneity was assessed using I² and τ². Prediction intervals were considered conceptually but not emphasized because the number of eligible studies per outcome was small [28].

A prespecified sensitivity analysis excluded Aronne 2024 because it used a randomized-withdrawal design rather than a standard treatment-initiation design. Funnel plot assessment was performed for the primary outcome only. Formal Egger testing was not performed because fewer than 10 studies were available, making asymmetry tests unreliable [29,30].

Risk of Bias Assessment

**Figure 2:**
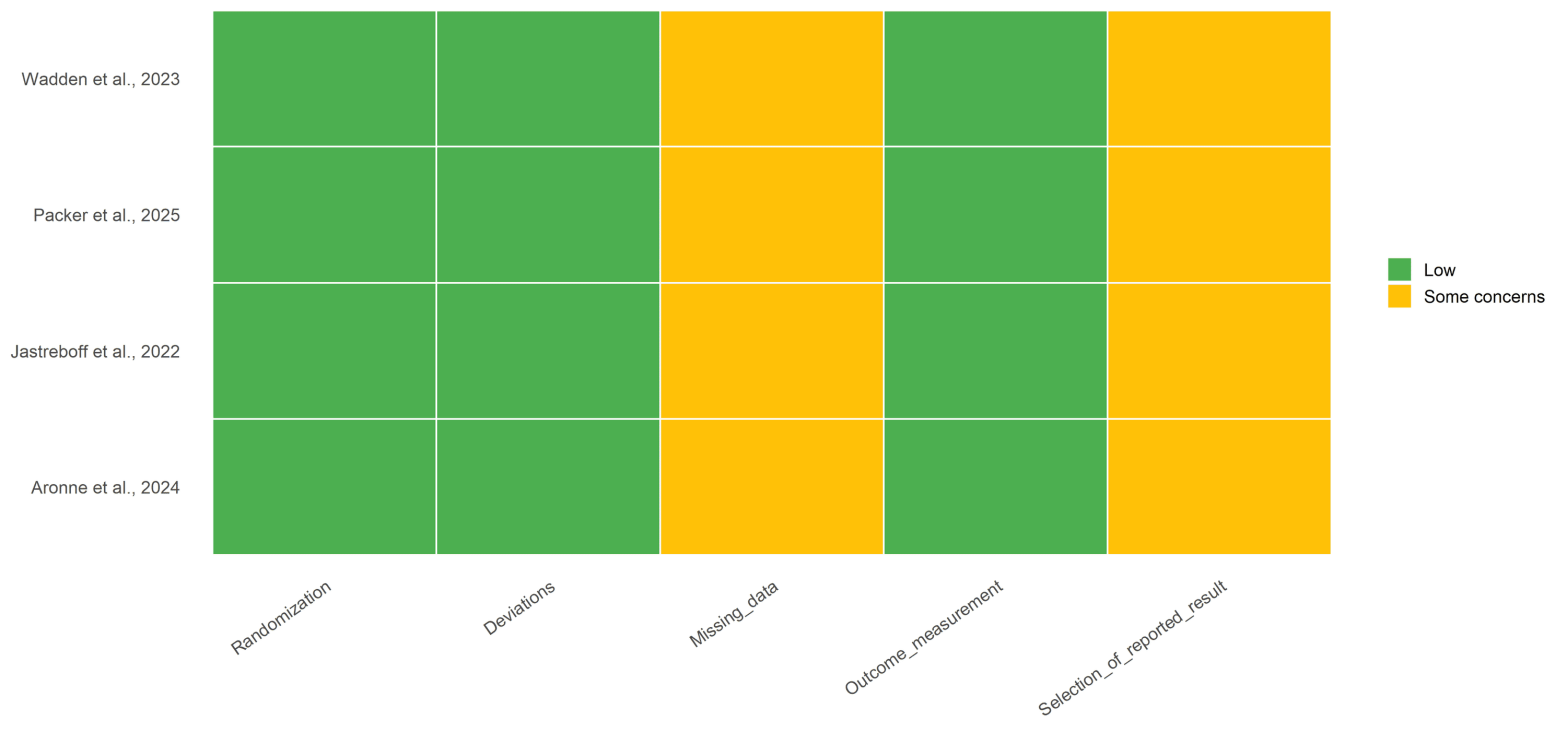
Risk-of-bias summary [16-19]

The risk of bias for RCTs was assessed using the Cochrane Risk of Bias 2 tool (Figure 2) [31]. The following domains were assessed: randomization process, deviations from intended interventions, missing outcome data, measurement of outcomes, and selection of reported results.

Based on the available trial designs, the main anticipated risks are not randomization failure but rather clinical heterogeneity, selective outcome extraction, and the randomized-withdrawal structure of Aronne et al.

Results

Study Selection

The main quantitative synthesis included four RCTs: Jastreboff et al. [16], Wadden et al. [17], Aronne et al. [18], and Zhao et al. [19]. Three additional studies were retained for narrative cardiovascular synthesis: Packer et al. [21], Zile et al. [22], and Wilson et al. [23].

T2DM-only or T2DM-dominant tirzepatide trials were excluded from the main pooled analysis because the review question focused on adults without diabetes [31-39]. Previous reviews, narrative reviews, and meta-analyses were not treated as primary studies [40].

In Table 2 [16-19], Jastreboff et al. [16] was the largest included RCT, randomizing 2539 adults without diabetes to tirzepatide 5 mg, 10 mg, 15 mg, or placebo. Wadden et al. [17] randomized 579 participants after a 12-week intensive lifestyle lead-in. Aronne et al. [18] randomized 670 participants after a 36-week open-label tirzepatide lead-in period and are therefore methodologically distinct from standard initiation trials. Zhao et al. [19] randomized 210 Chinese adults without diabetes to tirzepatide 10 mg, 15 mg, or placebo for 52 weeks.

**Table 2:** Characteristic of included studies RCT: randomized controlled trial

Study	Design	Population	Diabetes status	Intervention	Comparator	Follow-up	Main use
Jastreboff et al. (2022) [16]	Phase 3, double-blind RCT	Adults with obesity or overweight with a weight-related complication	Diabetes excluded	Tirzepatide 5 mg, 10 mg, or 15 mg	Placebo	72 weeks	Main pooled analysis
Wadden et al. (2023) [17]	Phase 3 RCT after intensive lifestyle lead-in	Adults with obesity/overweight who lost ≥5% with lifestyle intervention	Diabetes excluded	Tirzepatide maximum tolerated dose 10/15 mg	Placebo	72 weeks post-randomization	Main pooled analysis
Aronne et al. (2024) [18]	Randomized-withdrawal RCT	Adults with obesity/overweight after open-label tirzepatide lead-in	Diabetes excluded	Continued tirzepatide maximum tolerated dose	Switched to a placebo	88 weeks total	Main analysis and sensitivity flag
Zhao et al. (2024) [19]	Phase 3, double-blind RCT	Chinese adults with obesity/overweight and weight-related comorbidity	Diabetes excluded	Tirzepatide 10 mg or 15 mg	Placebo	52 weeks	Main pooled analysis

Primary Outcome: Percentage Body Weight Change

All four RCTs favored tirzepatide over placebo for the percentage change in body weight. The pooled random-effects estimate showed a significant and clinically large reduction in percentage body weight with tirzepatide compared with placebo:

$MD = -18.42\%; \ 95\% \ CI, \ -22.05 \ \mathrm{to} \ -14.79; \ I^{2} = 70.9\%$

This indicates that tirzepatide was associated with approximately an 18-percentage-point greater body weight reduction than the placebo. Although heterogeneity was substantial, the direction of effect was consistent across all included trials.

**Figure 3:**
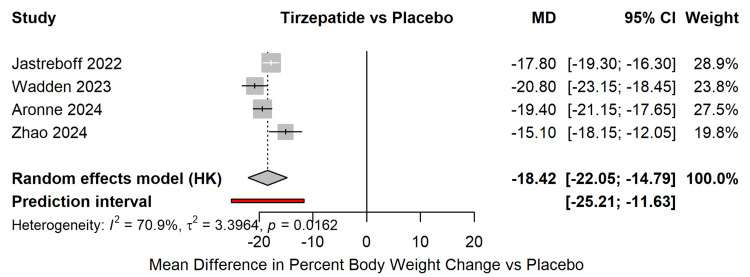
Forest plot of percentage body weight change MD: mean difference, CI: confidence interval [16-19]

Figure 3 shows a forest plot of the percentage change in body weight with tirzepatide versus placebo in adults who are obese or overweight without diabetes. Negative mean differences favor tirzepatide. The analysis used the 15 mg dose arm for fixed-dose trials and the maximum tolerated dose for dose-escalation trials.

Sensitivity Analysis Excluding Randomized-Withdrawal Trial

Because Aronne et al. [18] used a randomized-withdrawal design, a sensitivity analysis excluding this study was performed. The pooled effect remained large and directionally consistent:

$MD = -18.01\%; \ 95\% \ CI, \ -24.86 \ \mathrm{to} \ -11.16; \ I^{2} = 77.6\%$

**Figure 4:**
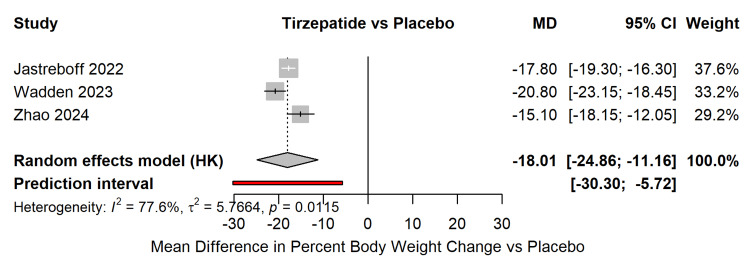
Sensitivity analysis MD: mean difference, CI: confidence interval [16-17,19]

This suggests that the primary body weight result was not driven solely by the withdrawal design. Figure 4 shows the sensitivity analysis. The pooled body weight effect remained strongly favorable to tirzepatide after excluding the randomized withdrawal trial.

Waist Circumference

Three trials contributed extractable data for waist circumference. Tirzepatide significantly reduced waist circumference compared with placebo:

$MD = -15.20 \ \mathrm{cm}; \ 95\% \ CI, \ -17.69 \ \mathrm{to} \ -12.70; \ I^{2} = 23.5\%$

**Figure 5:**
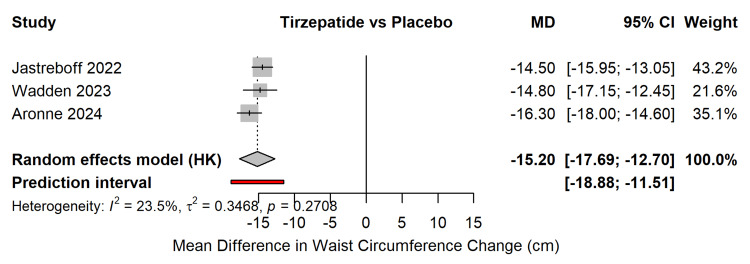
Forest plot of waist circumference MD: mean difference, CI: confidence interval [16-18]

This finding supports a substantial reduction in central adiposity. Heterogeneity was relatively low compared with the body weight analysis. Figure 5 shows the forest plot of the change in waist circumference with tirzepatide versus placebo. Negative mean differences favor tirzepatide.

Blood Pressure Outcomes

Two trials contributed extractable estimates for systolic and diastolic blood pressure. Both individual study estimates favored tirzepatide. However, pooled Hartung-Knapp confidence intervals were wide because only two trials were available. For systolic blood pressure:

$MD = -7.62 \ \mathrm{mmHg}; \ 95\% \ CI, \ -26.65 \ \mathrm{to} \ 11.41; \ I^{2} = 82.3\%$

For diastolic blood pressure:

$MD = -4.65 \ \mathrm{mmHg}; \ 95\% \ CI, \ -14.10 \ \mathrm{to} \ 4.79; \ I^{2} = 68.0\%$

These findings should be interpreted as directionally favorable but statistically imprecise. The evidence suggests a reduction in blood pressure, but the pooled estimates are fragile due to the small number of studies and heterogeneity.

**Figure 6:**
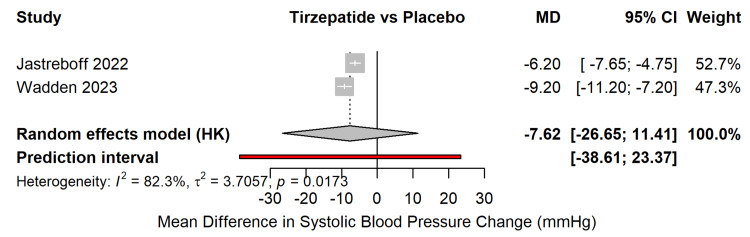
Forest plot of systolic blood pressure MD: mean difference, CI: confidence interval [16,17]

**Figure 7:**
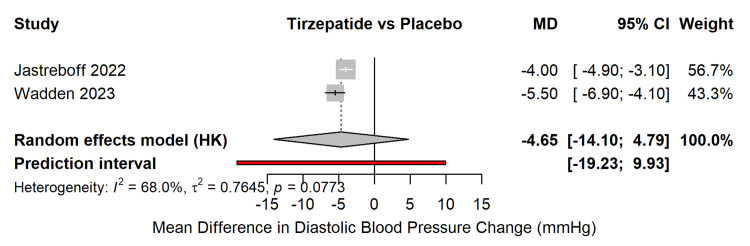
Forest plot of diastolic blood pressure MD: mean difference, CI: confidence interval [16,17]

Figure 6 shows the forest plot of the change in systolic blood pressure with tirzepatide versus placebo. Negative mean differences indicate greater reductions in systolic blood pressure with tirzepatide. Figure 7 shows the forest plot of the change in diastolic blood pressure with tirzepatide versus placebo. Negative mean differences indicate greater reductions in diastolic blood pressure with tirzepatide.

Lipid Outcomes

Two studies contributed extractable lipid percentage-change estimates. For triglycerides, both included studies favored tirzepatide:

$MD = -24.10\%; \ 95\% \ CI, \ -73.01 \ \mathrm{to} \ 24.82; \ I^{2} = 83.9\%$

For LDL cholesterol:

$MD = -7.75\%; \ 95\% \ CI, \ -54.11 \ \mathrm{to} \ 38.61; \ I^{2} = 87.9\%$

For HDL cholesterol:

$MD = +9.94\%; \ 95\% \ CI, \ -6.45 \ \mathrm{to} \ 26.32; \ I^{2} = 31.3\%$

The individual trial estimates favored improved lipid profiles with tirzepatide, but pooled inference was imprecise because only two studies were available for each lipid outcome. Therefore, these findings should be presented as supportive rather than definitive.

**Figure 8:**
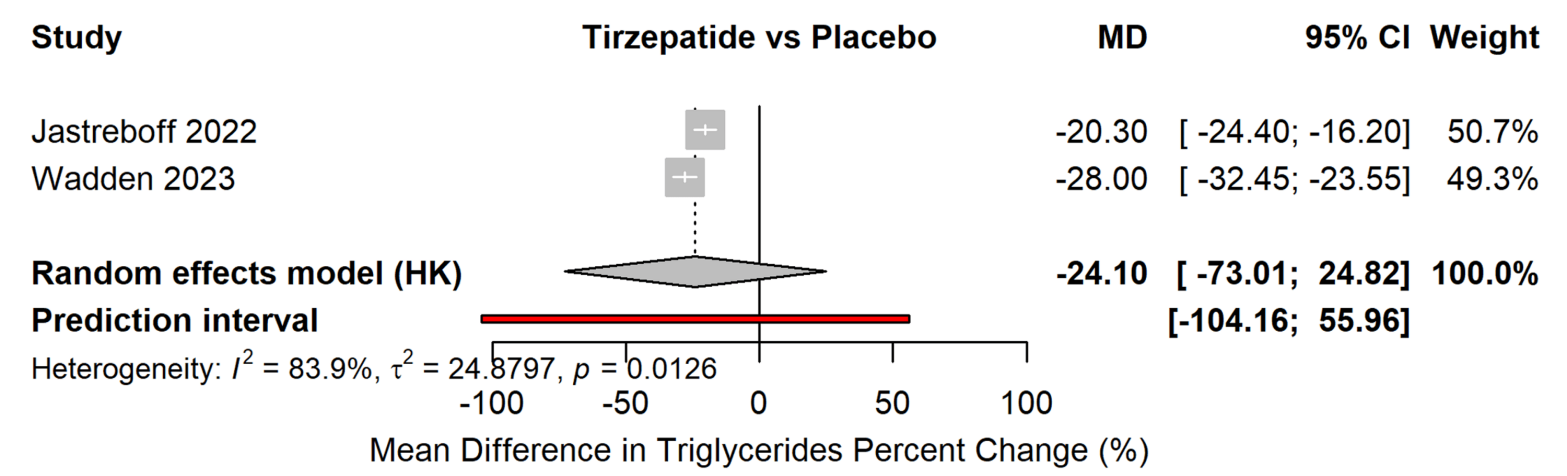
Forest plot of triglycerides MD: mean difference, CI: confidence interval [16,17]

**Figure 9:**
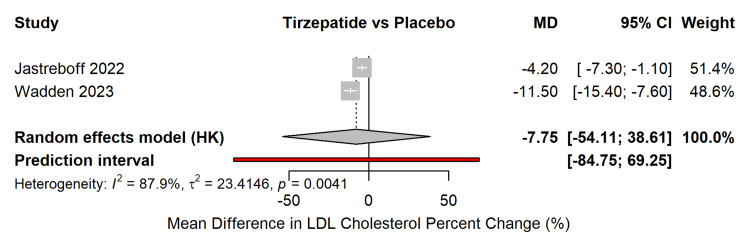
Forest plot of LDL cholesterol MD: mean difference, CI: confidence interval. LDL: low-density lipoprotein [16,17]

**Figure 10:**
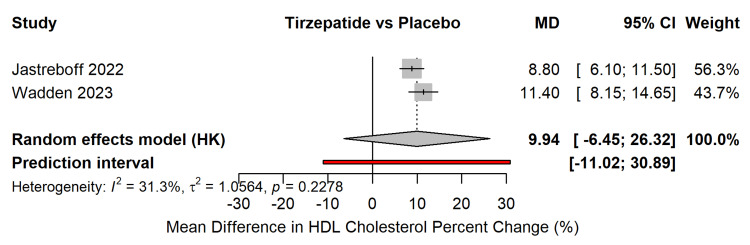
Forest plot of HDL cholesterol MD: mean difference, CI: confidence interval, HDL: high-density lipoprotein [16,17]

Figure 8 shows the forest plot of the percentage change in triglycerides with tirzepatide versus the placebo. Negative mean differences favor tirzepatide. Figure 9 shows the forest plot of the percentage change in LDL cholesterol with tirzepatide versus the placebo. Negative mean differences favor tirzepatide. Figure 10 shows the forest plot of the percentage change in HDL cholesterol with tirzepatide versus placebo. Positive mean differences favor tirzepatide because higher HDL cholesterol is generally favorable. Table 3 summarizes the pooled cardiometabolic outcomes.

**Table 3:** Summary of pooled cardiometabolic outcomes MD: mean difference, CI: confidence interval, BP: blood pressure, LDL: low-density lipoprotein, HDL: high-density lipoprotein

Outcome	Studies	Pooled MD	95% CI	I²	Interpretation
Body weight percent change	4	-18.42%	-22.05 to -14.79	70.9%	Strongly favors tirzepatide
Body weight sensitivity excluding Aronne et al. (2024)	3	-18.01%	-24.86 to -11.16	77.6%	Effect remains robust
Waist circumference	3	-15.20 cm	-17.69 to -12.70	23.5%	Strongly favors tirzepatide
Systolic BP	2	-7.62 mmHg	-26.65 to 11.41	82.3%	Direction favors tirzepatide, imprecise
Diastolic BP	2	-4.65 mmHg	-14.10 to 4.79	68.0%	Direction favors tirzepatide, imprecise
Triglycerides	2	-24.10%	-73.01 to 24.82	83.9%	Direction favors tirzepatide, imprecise
LDL cholesterol	2	-7.75%	-54.11 to 38.61	87.9%	Direction favors tirzepatide, imprecise
HDL cholesterol	2	+9.94%	-6.45 to 26.32	31.3%	Direction favors tirzepatide, imprecise

Publication Bias

**Figure 11:**
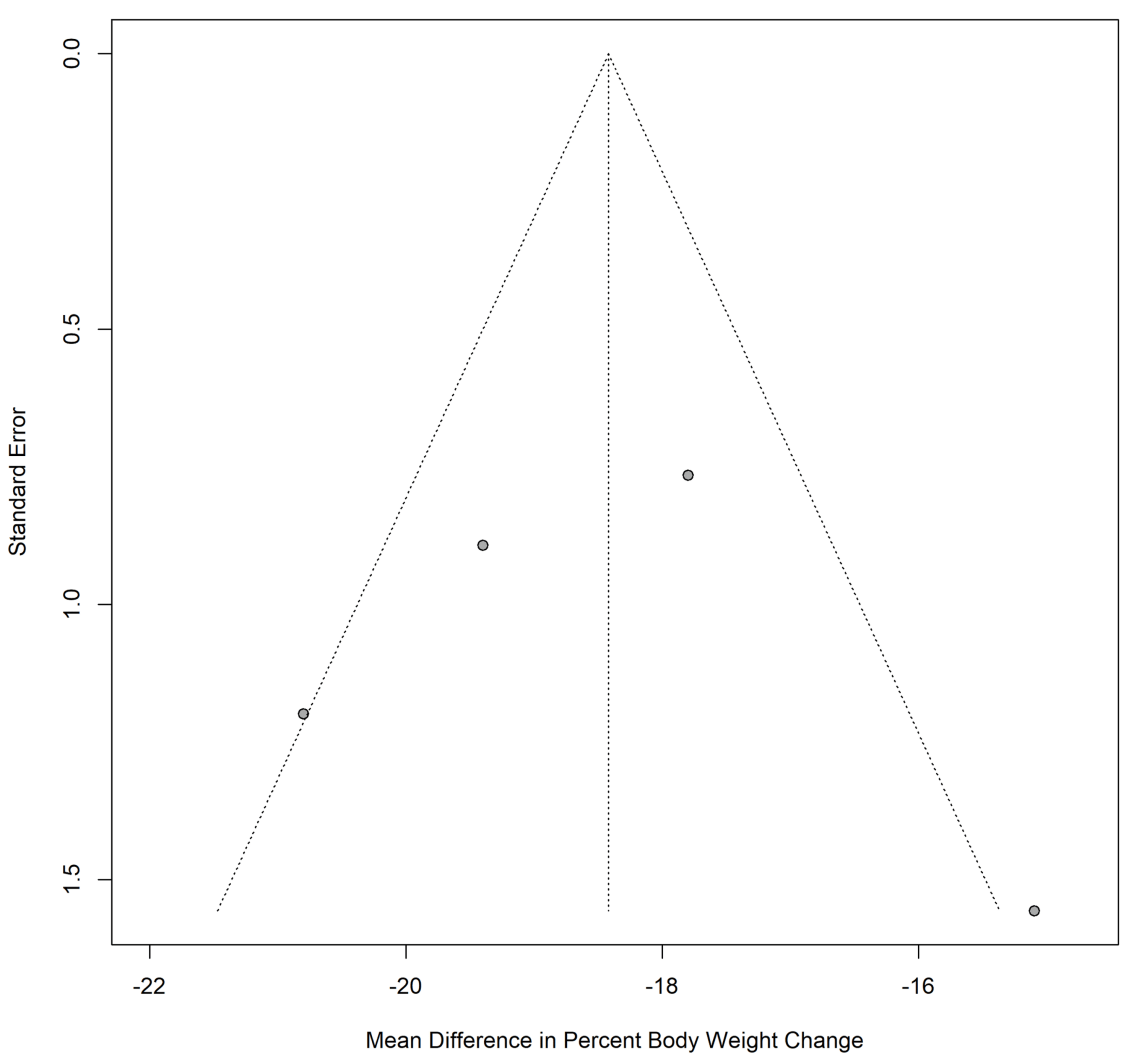
Funnel plot for percentage body weight change

A funnel plot was generated for the primary outcome of percentage body weight change. Interpretation is limited because only four trials were included. Formal funnel plot asymmetry testing was not performed because fewer than 10 studies were available [29,30]. Figure 11 shows the funnel plot for percentage body weight change. The small number of included studies limits the interpretation of the funnel plot.

Narrative Cardiovascular Outcomes Synthesis

Evidence regarding cardiovascular outcomes in adults with obesity without diabetes remains limited because most available data come from HFpEF or observational cohorts rather than primary obesity RCTs [20-23].

In Table 4, Packer et al. [21] explored the effects of tirzepatide on cardiovascular events in HFpEF with obesity. Treatment resulted in lower composite rates of cardiovascular death or heart failure hospitalization compared with placebo and better health status (as measured by the Kansas City Cardiomyopathy Questionnaire clinical summary score). Still, this trial focused on an HFpEF cohort in which nearly half of the subjects had T2DM. Thus, it cannot be pooled with other adult non-diabetic obesity trials.

**Table 4:** Cardiovascular evidence kept outside the main meta-analysis RCT: randomized controlled trial, HFpEF: heart failure with preserved ejection fraction, KCCQ-CSS: Kansas City Cardiomyopathy Questionnaire Clinical Summary Score, ASCVD: Atherosclerotic Cardiovascular Disease, rMACE: Revised Major Adverse Cardiovascular Events, T2DM: type 2 diabetes mellitus

Study	Design	Population	Main finding	Why not pooled
Packer et al. (2025) [21]	RCT	HFpEF + obesity	Reduced cardiovascular death/worsening heart failure composite; improved KCCQ-CSS	HFpEF population; many had T2DM
Zile et al. (2025) [22]	Secondary SUMMIT analysis	HFpEF + obesity	Improved clinical trajectory and functional outcomes	Same trial population as Packer et al. (2025)
Wilson et al. (2026) [23]	Observational cohort	Overweight/obesity + ASCVD without diabetes	Semaglutide is associated with lower rMACE versus tirzepatide	Observational; active comparator

Zile et al. [22] further analyzed the SUMMIT trials. They demonstrated tirzepatide effects on a wide range of clinical trajectory domains, including health status, functional capacity, quality of life, exercise tolerance, patient well-being, NYHA class, and medication burden. While it provides valuable evidence for the HFpEF subgroup within the obesity population, it does not demonstrate any general effect on MACE reduction in non-diabetic obesity.

Wilson et al. [23] investigated real-world cardiovascular outcomes in an observational cohort of overweight/obese individuals with ASCVD, but without diabetes. It appeared that semaglutide treatment led to a decrease in both rMACE-3 and rMACE-5 compared with tirzepatide treatment. Although these findings can be considered clinically relevant, there is no way to establish randomized superiority due to the observational design, the use of propensity score matching, and the comparison of semaglutide to tirzepatide rather than to a placebo.

Therefore, although some positive findings have been observed in certain patient subgroups, evidence on cardiovascular outcomes in adults with obesity without diabetes is insufficient to claim a reduction in MACE.

Discussion

This systematic review and meta-analysis demonstrated significant reductions in body weight and waist circumference in adults with overweight or obesity without diabetes [16-19]. Body weight analysis suggested an 18-percentage-point reduction in body weight with tirzepatide treatment. Importantly, the effect seemed to remain robust even after a sensitivity analysis that removed Aronne et al., thus eliminating concerns about possible study design influence on the overall findings [18].

A pooled reduction in waist circumference by 15 cm is particularly interesting, given the relationship between waist circumference and cardiometabolic risk factors. Indeed, central adiposity is positively associated with such risk factors as insulin resistance, increased inflammatory burden, NAFLD, elevated blood pressure, and abnormal lipid metabolism [3-6]. The low level of heterogeneity observed for waist circumference also speaks to the consistency of findings.

Favorable effects of tirzepatide on blood pressure and lipids were directionally consistent; however, they cannot be treated as statistically robust evidence. It should be emphasized that this is not due to a lack of biological effect of tirzepatide but rather because only two trials provided extractable evidence for these outcomes. Importantly, in both included studies, blood pressure, triglycerides, LDL, and HDL concentrations were shown to favor tirzepatide treatment. Still, very wide H-K confidence intervals, along with high heterogeneity, limit the statistical value of findings.

The significance of body weight reduction observed in the current meta-analysis should not be underestimated. A reduction of 5-10% can help manage many obesity-related comorbidities; in turn, higher reductions are usually needed to reduce obstructive sleep apnea, NAFLD, hypertension, and heart failure risks [5-9]. Given the current pooled reduction exceeding this threshold, tirzepatide appears to have a strong effect on the cardiometabolic risk profile of adults with obesity.

The proposed mechanism of action for tirzepatide seems to be multifactorial [13-15]. Its effects on weight loss stem from its ability to suppress appetite and caloric intake via incretin pathways. On the other hand, weight loss reduces cardiac load, blood pressure, visceral fat deposition, inflammatory burden, and insulin resistance. Effects of glucagon-like peptide-1 receptor activation include natriuresis, anti-inflammatory properties, and endothelial effects. In turn, GIP receptor activity impacts adipose tissue function [13-15].

It should be noted that the interpretation of cardiovascular evidence requires caution. While there is convincing evidence for improvements in cardiometabolic risk factors, there is no evidence that demonstrates the reduction in hard cardiovascular events in the non-diabetic obesity RCT population. Although Packer et al. [21] and Zile et al. [22] provide valuable evidence in the context of HFpEF, it cannot be applied to the non-diabetic obese population, as these studies included older, more complex patients, many of whom had T2DM. Also, Wilson et al. [23] provide valuable evidence for ASCVD in adults without diabetes. Nevertheless, this trial has an observational design and suggests that semaglutide is superior to tirzepatide in early MACE outcomes. Therefore, it would be misleading to conclude that tirzepatide leads to a reduction in cardiovascular events. Rather, it should be emphasized that the drug provides considerable cardiometabolic benefits, while evidence for cardiovascular outcomes is still evolving.

Strengths

This review has several strengths. First, it focused exclusively on adults with obesity or overweight and excluded patients with T2DM. Second, the inclusion criteria separated placebo-controlled obesity RCTs from HFpEF and observational cardiovascular trials [16-23]. Third, author-year notation was used for trials included in the analysis. Fourth, a sensitivity analysis excluding Aronne 2024 [18] was employed to address the issue of randomized-withdrawal design.

Limitations

However, this review has some weaknesses. First, the number of eligible RCTs was limited. Second, not all outcomes were extractable from all included studies. Blood pressure and lipid analysis involved just two trials. Third, Aronne et al. [18] employed a random-withdrawal design, which differs from a standard placebo-controlled design. Fourth, individual participant data were unavailable. Fifth, a publication bias test was not conducted, as there were fewer than 10 eligible studies [29,30]. Finally, cardiovascular outcomes could not be pooled due to differences in patient characteristics, comparators, and study designs [20-23].

Clinical Implications

Based on the results of this meta-analysis, one can state that tirzepatide leads to a significant reduction in body weight and waist circumference in adults who are obese or overweight and without diabetes [16-19]. These changes will likely improve cardiometabolic risk factors. At the same time, it is important to avoid overstating the cardiovascular event-reducing claims in patients with non-diabetic obesity until hard outcome trials are conducted [20-23]. Tirzepatide can be safely described as a powerful cardiometabolic agent for obesity, but it cannot be called a proven MACE-reducing therapy.

Future Research

Future trials should directly evaluate cardiovascular outcomes in adults with obesity but without diabetes, including MACE, cardiovascular mortality, myocardial infarction, stroke, heart failure hospitalizations, renal outcomes, and the durability of benefit after long-term treatment. Beyond hard endpoints, future studies should also incorporate imaging, biomarker, and inflammatory marker assessment to clarify mechanisms of benefit. Cardiac MRI, echocardiography, coronary CT angiography, and body-composition imaging may help assess changes in left ventricular mass, ventricular function, visceral adiposity, liver fat, epicardial or paracardiac adipose tissue, and coronary plaque burden. This is important because epicardial adipose tissue is metabolically active and linked to myocardial and coronary inflammatory signaling. At the same time, SUMMIT imaging data suggest that tirzepatide may reduce left ventricular mass and pericardiac adipose tissue in obesity-related HFpEF. Biomarkers such as NT-proBNP, high-sensitivity troponin, lipid fractions, renal markers, insulin-resistance indices, high-sensitivity C-reactive protein, interleukin-6, TNF-α, adiponectin, and leptin may help distinguish benefits mediated by weight loss from those related to incretin-specific effects, adipose tissue remodeling, and reduced systemic inflammation.

## Conclusions

Tirzepatide significantly reduced body weight and waist circumference in adults with obesity or overweight and without diabetes. Favorable effects on blood pressure and lipid parameters were observed, but evidence remains limited because few studies reported extractable data. Cardiovascular findings from HFpEF and observational cohorts are encouraging, but they cannot be pooled with the main obesity RCTs because of differences in populations, comparators, and study designs. Therefore, hard cardiovascular outcome trials in non-diabetic adults with obesity are still needed before claiming MACE reduction.

This review is distinct because it focuses specifically on non-diabetic obesity, separates placebo-controlled obesity RCTs from HFpEF and observational cardiovascular studies, and provides a cautious interpretation of tirzepatide’s cardiometabolic benefits without overstating cardiovascular event reduction.
